# Pre-, Pro-, and Synbiotics: Do They Have a Role in Reducing Uremic Toxins? A Systematic Review and Meta-Analysis

**DOI:** 10.1155/2012/673631

**Published:** 2012-12-19

**Authors:** Megan Rossi, Kerenaftali Klein, David W. Johnson, Katrina L. Campbell

**Affiliations:** ^1^School of Medicine, The University of Queensland, Brisbane, QLD 4006, Australia; ^2^Department of Nutrition and Dietetics, Princess Alexandra Hospital, Brisbane, QLD 4102, Australia; ^3^Department of Nephrology, Princess Alexandra Hospital, Brisbane, QLD 4102, Australia; ^4^Queensland Clinical Trials & Biostatistics Centre, School of Population Health, The University of Queensland, Brisbane, QLD 4006, Australia

## Abstract

*Objective*. This paper assessed the effectiveness of pre-, pro-, and synbiotics on reducing two protein-bound uremic toxins, p-cresyl sulphate (PCS) and indoxyl sulphate (IS). *Methods*. English language studies reporting serum, urinary, or fecal PCS and/or IS (or their precursors) following pre-, pro-, or synbiotic interventions (>1 day) in human adults were included. Population estimates of differences in the outcomes between the pre- and the postintervention were estimated for subgroups of studies using four meta-analyses. Quality was determined using the GRADE approach. *Results*. 19 studies met the inclusion criteria, 14 in healthy adults and five in haemodialysis patients. Eight studies investigated prebiotics, six probiotics, one synbiotics, one both pre- and probiotics, and three studies trialled all three interventions. The quality of the studies ranged from *moderate* to *very low*. 12 studies were included in the meta-analyses with all four meta-analyses reporting statistically significant reductions in IS and PCS with pre- and probiotic therapy. *Conclusion*. There is a limited but supportive evidence for the effectiveness of pre- and probiotics on reducing PCS and IS in the chronic kidney disease population. Further studies are needed to provide more definitive findings before routine clinical use can be recommended.

## 1. Introduction

Chronic kidney disease (CKD) coupled with cardiovascular disease (CVD) is emerging as a major public health problem. CKD has become one of the most common chronic conditions attributable to the burden of disease worldwide [1]. Moreover, CVD is highly prevalent in CKD, such that CKD patients are far more likely to experience cardiovascular (CV) mortality than progression to end-stage renal failure [2]. Treatment to reduce both CKD progression and CV mortality is urgently required. This paper investigates a potential therapeutic strategy targeting the gut with a low cost, innovative nutrition-based treatment of pre- and probiotics.

Recent studies suggest that two protein-bound toxins implicated in the uremic syndrome, p-cresyl sulphate (PCS) and indoxyl sulphate (IS), may be risk factors for the high CV mortality rates observed in the CKD population [3–6]. Both PCS and IS originate exclusively from dietary amino acid bacterial fermentation in the large intestine [7]. CKD enhances the protein fermentation process through a number of mechanisms including inefficient protein assimilation in the small intestine resulting in more protein entering the large intestine, prolonged colonic transit time, and increased luminal pH secondary to increased colonic urea diffusion, all of which contribute to the alteration of the bacterial composition of the microbiota (colonic microenvironment) specific to this population [8, 9]. This increase in PCS and IS toxin production in CKD patients, coupled with inadequate renal clearance, results in high serums levels, which are inversely correlated with glomerular filtration rate [10]. 

Lowering the production of these uremic toxins by manipulating bacterial composition of the microbiota and/or colonic transit time therefore represents a promising therapeutic strategy. Pre- and probiotics may be able to facilitate such a change in the colonic environment by enhancing the ratio of available carbohydrates to nitrogen, increasing short-chain fatty acid production, decreasing colonic pH, increasing colonic transit time, and repressing the enzymes which catalyse the reactions producing PCS and IS. Therefore, in theory, this treatment offers compelling therapeutic appeal.

The objective of this paper is to assess the effectiveness of pre-, pro-, and synbiotics on reducing PCS and IS production. A secondary aim is to identify the most effective intervention, including probiotic strain/prebiotic variety, dosage, and duration, as well as to highlight the gaps in the literature to guide future studies in this area.

## 2. Methods

**Figure 1:**
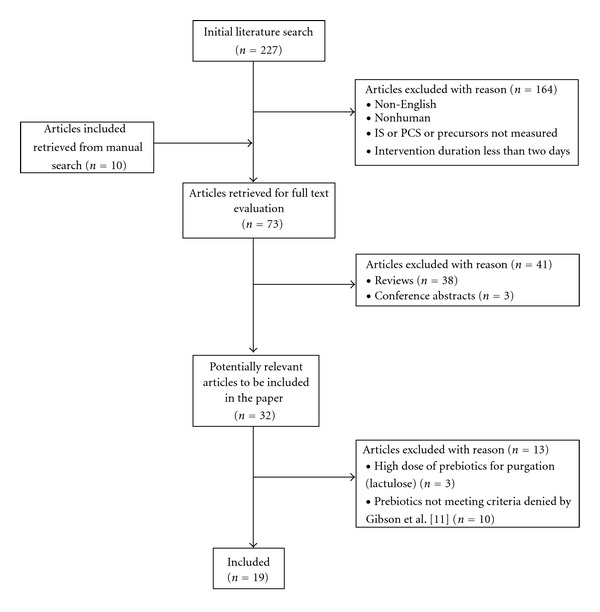
Search methodology for the PCS and IS literature review.

An extensive review of the literature from 1951 to 2011 (inclusive) was conducted using Cochrane, PubMed, Embase, and CINAHL. A combination of the following Medical Subject Heading search terms were used: Prebiotics, Probiotics, Synbiotics, Oligosaccharides/therapeutic, Fructans/therapeutic use, Bacteria/therapeutic use and Kidney Diseases, Indican, Cresols, Tyrosine/metabolism, Tryptophan/metabolism. In addition, manual searches were performed to identify studies and literature reviews from the bibliographies of relevant published articles. A search limited to English language studies of human adults, that had implemented a pre-, pro-, or synbiotic intervention for longer than one day resulted in 237 articles (including 10 studies identified from the manual search). After applying the exclusion criteria (review articles, studies which did not report PCS and/or IS (or their precursors), use of nonvalidated prebiotics as defined by Gibson et al. [11], and studies using high doses of prebiotics for purgation (i.e., lactulose)), only 19 remained.  Figure 1 indicates a flow diagram of the methodology.

Studies were classified using the study design definitions from the National Health and Medical Research Council [12]. The Grades of Recommendation, Assessment, Development, and Evaluation (GRADE) approach for grading the evidence was applied [13]. 

The unit of measurement for PCS and IS was converted to milligrams (mg)/litre (L) (serum), mg/day (urine), or mg/gram (g) (fecal) where possible using molecular weights from the Human Metabolome Database [14]. P-cresol (PC), an artefact of PCS induced by hydrolysis in the analytical process, is used interchangeably with PCS in this paper [15]. Also, indole is the precursor for IS prior to hydroxylation and sulphation in the body. Therefore, fecal indole is a surrogate marker of urinary and serum IS.

Attempts were made to contact corresponding authors for information that was not published, including probiotic form (i.e., powder or capsule) and strain, analysis information, missing numerical values of outcome measures, and information on dietary protocol.

### 2.1. Statistical Analysis

The summary statistics reported from the relevant studies were translated into means and standard deviations (SD), assuming normal distribution. SDs were obtained via simulation for those studies which reported median and interquartile range. 

The meta-analysis was performed to estimate the true population treatment effect and to find factors significantly associated with the outcome variable, which was the mean change in serum IS/urinary PCS before/after treatment. The meta-analysis was limited by the number of studies that could be included given the inconsistencies in the outcome measures reported, that is, serum, urine, and fecal. Four meta-analyses were undertaken. The first analysis looked at serum IS as an outcome from pre-, pro-, and synbiotic interventions in the HD population. The other three meta-analyses were in the healthy population investigating urinary PCS as the outcome. One of the three from the healthy population included only prebiotic interventions, another solely probiotics, and the final a combination of all three: pre-, pro-, and synbitoic interventions.

Fixed- and random-effects meta-analyses were performed where the random-effects model was fitted with restricted maximum-likelihood estimators for the amount of heterogeneity. The best model fit was selected using the likelihood ratio test. Potential publication bias was investigated using funnel plots. The significance of between-trial heterogeneity was also tested. Sensitivity analyses were undertaken to determine any possible effects relating to the assumed correlation between pre- and post- IS/PCS measures to the estimated true population treatment effect. 

Possible associations of treatment dose and types of pre- and probiotics with the outcomes were investigated using multivariable metaregression. Akaike and Bayesian information criteria were used to determine whether the models with the covariates fitted the data significantly better. R program (version 2.14.0) [16] with metaphor library [17] was used to perform the meta-analysis and meta regression.

## 3. Results

### 3.1. Literature Review

The 19 eligible studies consisted of 14 studies in the healthy population [18–31] and five studies in patients with kidney disease [32–36], all of whom were undergoing haemodialysis (HD) for end-stage kidney disease (ESKD). Seven studies conducted in the healthy and one from the HD population investigated prebiotics (Table 1); [18–24, 32] six trialled probiotics, three in the healthy [25–27] and the others in the HD population [33–35] (Table 2); one looked at both pre- and probiotics separately, [29] one studied synbiotics alone [36], and three studies investigated the effects of all three types of interventions (Table 3) [28, 30, 31]. 

There was one randomised placebo-controlled trial [31], four randomised placebo-controlled crossover studies [18, 28–30], three nonrandomised placebo-controlled experimental trials [24, 25, 33], two interrupted time series without a parallel control group [26, 36], and 9 case series [19–23, 27, 32, 34, 35]. 

### 3.2. Validity

The overall quality of the studies that met the inclusion criteria was limited. The highest grade in this paper was *moderate* which four papers achieved [18, 25, 28, 29], eight papers were *low *[19–22, 24, 27, 30, 33], and the other seven papers were classified as *very low* (Tables 1, 2, and 3). 

#### 3.2.1. Diet

Overall, only one study involved a dietitian to ensure monitoring and controlling for dietary changes [24]. Nine studies made some attempt to control for the diet, such as encouraging the participants to maintain a “stable” or “regular” diet and advised a range of food restrictions, including fermented products, pickles, and natto (traditional Japanese dish) during the intervention [18–22, 27–30]. Four studies made an attempt to monitor the participants' diets throughout the study [23–25, 31]. One study that monitored fibre intake found that the probiotic group had 10 g more fibre than the prebiotic group [31]. This study subsequently found no significant change in uremic toxin levels between the two interventions.

#### 3.2.2. Outcome Measures

The 19 studies spanned across the past 30 years, utilising a range in analytical techniques, therefore making it difficult to directly compare results across studies. The source of outcome measures of PCS and IS ranged from serum concentrations in the HD population to urinary and fecal excretion in the healthy population. Within the studies that reported fecal measures, there were nonconvertible differences between units, that is, mg/g of dry fecal weight, mg/g wet fecal weight and mg/L. 

The range in outcome measures was a limitation to which studies were included in the meta-analyses.

### 3.3. Prebiotic Studies 

From the 19 studies that met the inclusion criteria, there were 13 interventions that used prebiotics [18–24, 28–32], with only one in the HD population (Tables 1 and 3) [32]. Twelve of these interventions observed a trend for a decrease in PCS and/or IS, but only eight of 11 reported a significant decrease in PCS and three out of five a significant decrease in IS.

#### 3.3.1. Meta-Analysis

Six out of the 11 studies looking at PCS postintervention were included in the meta-analysis [18, 19, 23, 28–30] with the exclusion of one study in the HD population [32] and five that measured fecal PCS [20–22, 24, 31]. The meta-analysis included five interventions from randomised placebo-controlled crossover trials [18, 28–30] and two case series [19, 23]. The studies used a range of prebiotics and doses, arabinoxylan-oligosaccharide (AXOS) 10 g, [18] oligofructose-enriched inulin (OF-IN) 20 g [19, 30], inulin 15 g [23], and lactulose 20–30 g [19, 28, 29], with a total of 136 patients.

 The estimated population treatment effect size in urinary PCS was −7.4 mg/day (95% CI: 5.8–9.0). Meta regression was performed using both dose and type of prebiotics as covariates. The model with the type of treatment as the covariate performed significantly better. However, the estimated true population treatment effects did not differ between the models with/without the type of treatment.

#### 3.3.2. Other Studies

Three out of the five studies that utilised fecal analysis to assess changes in PC and/or indole production found a significant decrease in indole concentrations [20–22] and a trend for reduced PC concentration, that only reached statistical significance in one study [20]. Both of the studies which found a nonsignificant decrease in PC used considerably smaller doses of the prebiotic (3 g lactulose [22], 2.5 g GOS [21]) compared to the other positive studies.

The single prebiotic study conducted in the HD population (*n* = 22) was a case series design and looked at the effect on both serum IS and PCS [32]. Following the administration of 20 g of OF-IN for four weeks, there was a significant reduction in PC, and a trend for a decrease in IS. 

#### 3.3.3. Effect on Microbiota

Six studies citing eight interventions looked at the prebiotic effect on the microbiota, of which seven reported an increase in either bifidobacteria [18, 19, 21, 30] or both *Bifidobacterium* and *Lactobacillus* [20, 22]. None of the studies saw a change in the total number of bacteria [18, 20–22], and three studies identified that the changes seen in the microbiota were diminished after the prebiotic was discontinued [18, 20, 21].

The study by Cloetens et al. [18] reported a significant inverse correlation between the levels of bifidobacteria before prebiotic intake and the change in bifidobacterium levels after the three-week intervention [18]. 

The one negative study, which did not find a significant difference in bifidobacteria, also did not observe a change in fecal indole concentration [24]. 

#### 3.3.4. Effect on Colonic Transit Time and Fecal Characteristics

Four studies, all from in the healthy population, measured the oral-caecal transit time before and after prebiotic intervention using either labelled substrate method [18, 19, 23] or carboxylic acid breath test [28]. None found a significant change.

One study in the HD population reported an increase in stool quantity following synbiotic treatment, with no change in frequency, form or ease of defecation [36]. Importantly, over 70% of the study population was on regular laxatives throughout the study. None of the other six studies which measured fecal characteristics found a change in fecal weight, consistency, or frequency in the healthy population.

#### 3.3.5. Longevity of Prebiotic Effect

Out of nine studies that found a significant decrease in PC and/or IS, seven measured the run-out effect [18, 20–22, 28–30, 32]. Only two of these studies reported a continued difference after the intervention, day six [21] and week four [32] postintervention. The other studies reported a return to preintervention values as early as the day following the intervention [18, 29, 30].

#### 3.3.6. Gastrointestinal Tolerance

Three studies measured the tolerability of the prebiotic dose through a Likert questionnaire[18, 32] and a symptom diary [24]. The dose of prebiotics ranged from 20 g of OF-IN [32], 10 g AXOS [18], and 7.5–15 g TOS [24] divided into at least two half doses over the day. Flatulence was recognised by all three studies as a symptom. However, the two studies which collected quantitative outcomes reported a mild grade flatulence [18] and only 15% reported a negative tolerance overall [32]. 

### 3.4. Probiotic Studies

There were 11 interventions that administered probiotics using an array of different species and strains [25–31, 33–35]. Nine of these studies saw a decreasing trend in PCS and/or IS postintervention, which all seven and four from five found significance, respectively. 

#### 3.4.1. Meta-Analysis

Four out of the seven studies looking at PCS postintervention were included in the meta-analysis [25, 28–30] with the exclusion of one study in the HD population [35] and two that did not measure urinary PCS in mg/day [26, 27].

All of the studies included were from placebo-controlled crossover trials, [25, 28–30] and included 83 participants in total. The five interventions investigated probiotics from two different domains bacteria, and yeast.

The population treatment effect size in urinary PCS was −3.95 mg/day (95% CI: −0.12, 8.02), although this reduction was not significant. When adjusted by probiotic type, the effect size seen with bacterial probiotics was significantly greater (−7.05 mg/day; 95% CI: 3.51, 10.58).

#### 3.4.2. Other Studies

Five of the six studies, not included in the meta-analysis, found a reduction in either PC and/or IS following probiotic intervention [26, 27, 33–35]. Tohyama et al. [26] also investigated mechanistic changes in gut activity and found a strong correlation (*r* = 0.93) between fecal tryptophanase activity and urinary IS, which also decreased postintervention. Hida et al. [35] investigated change in fecal flora which showed a significant decrease in the number of enterobacteria; however, no change in total number of bacteria was observed.

Five studies reported a run-out period and all of them found that PC and/or IS levels had returned to preintervention levels at two weeks postintervention [25–27, 30, 33].

### 3.5. Synbiotic Studies

Four studies assessed the effect of synbiotics on PCS [28, 30] and both PCS and IS [31, 36] concentrations. Three of these studies reported a trend for a decrease in PCS/IS, although only two of these reached significance with PCS [30, 36].

De Preter et al. conducted a randomized placebo-controlled crossover study which coadministered *Lactobacillus casei* Shirota 2 × 10^9^ and OF-IN: 20 g and found a significant difference in urinary PCS [30]. This study also looked at the effect on the microbiota whereby there was a trend for a greater increase in bifidobacteria in the synbiotic arm (subgroup *n* = 9) compared to the prebiotic arm (*n* = 19), although this did not reach statistical significance. Nakabayashi et al. administered the probiotics in powder form and at a lower dose compared to other studies and still found a significant decrease in PCS (no change in IS) [36]. Of note, this study had a small sample size, *n* = 7, and included a participant that continued taking a lactic acid bacteria-based medication throughout the study.

### 3.6. Summary Meta-Analysis

**Figure 2:**
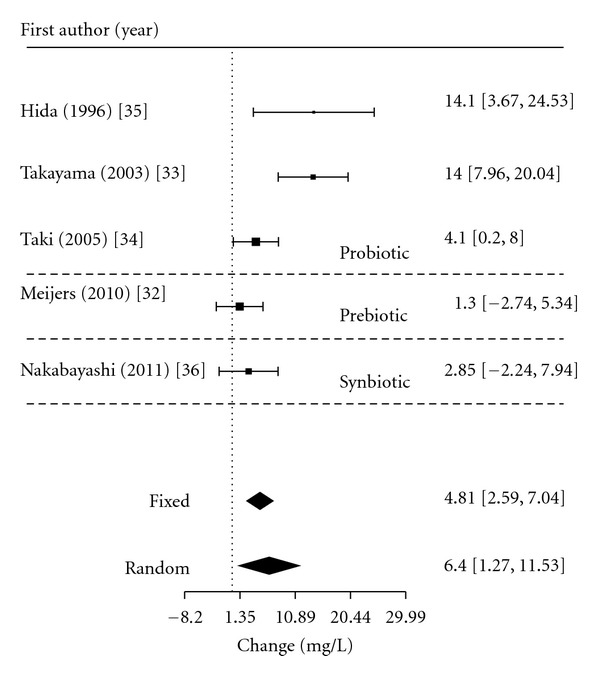
Meta-analysis for pre-, pro-, and synbiotic therapy on serum IS in the HD population.

**Figure 3:**
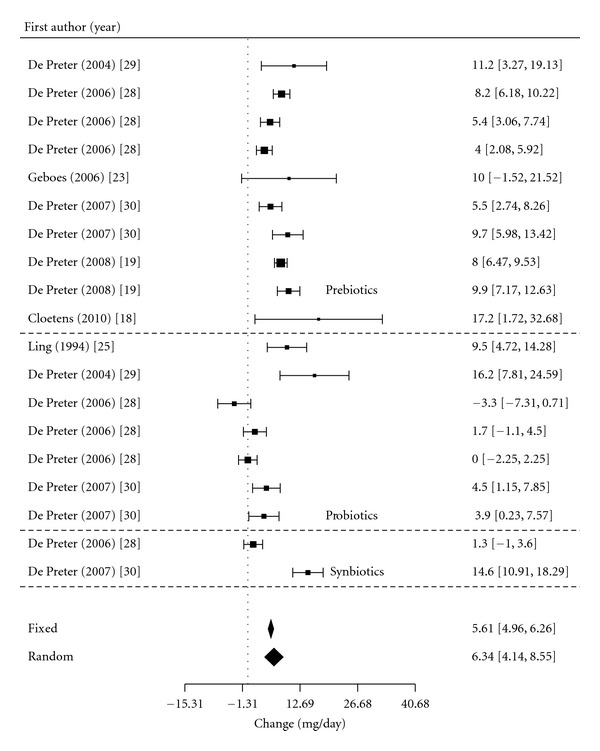
Meta-analysis for pre-, pro-, and synbiotic therapy on urinary PCS in the healthy population.

Two of the meta-analyses collated the effects of all interventions, pre-, pro-, and synbiotic studies, one looking at serum IS in the HD population (*n* = 87) (Figure 2) and the other at urinary PCS in the healthy population (*n* = 243) (Figure 3). Figure 2 demonstrates that the interventions in the HD population had a population effect size on serum IS of 6.4 mg/L (95% CI: 1.3 to 11.5). Given the limited number of relevant studies, meta regression was not able to be performed using the results from these studies. 

Figure 3 included all three treatments in the healthy population reported a population effect size of 6.34 mg/day (95% CI: 4.1, 8.6) in urinary PCS. Similarly to the prebiotic meta regression, when adjusted by type of intervention, the model performed significantly better despite no difference between the treatment effects.

### 3.7. Model Evaluation and Validation

Random effects models fitted the data best for all meta-analyses and the sensitivity analyses confirmed that the correlation assumptions (0.3–0.95) did not make clinically meaningful differences in the population outcome estimates. Hence, the correlation that best aligned with the published results was selected to be reported, 0.8 for the HD analysis and 0.95 for the healthy population. 

Funnel plots indicated a small degree of publication bias with small studies tending to report only positive findings in comparison to the large studies which reported both. The heterogeneity of each of the meta-analyses was statistically significant (*P* < 0.01) though this was reduced when the models were adjusted by type of treatment. 

## 4. Discussion

Overall there appeared to be a positive benefit of all three types of interventions, pre-, pro-, and synbiotics, on reducing the production of both PCS and IS. This benefit was seen in both the HD and healthy populations, although there was insufficient evidence to determine whether one treatment was more beneficial than the other. 

Thirteen interventions investigated prebiotics and all but one saw a trend for a decrease in PCS and/or IS [31] with the meta-analysis reported an overall decrease in urinary PCS by 7.4 mg/day. Of the 11 probiotic interventions nine reported a trend for a decrease in PCS and/or IS, with the meta-analysis depicted an overall decrease in urinary PCS by 7.05 mg/day following bacterial probiotic therapy. Out of the four synbiotic interventions, three reported a trend for a decrease in PC and/or IS [28, 30, 36] with only two reaching statistical significance for PC [30, 36]. 

The studies investigating synbiotics were sparse and achieved variable results. The standout synbiotic study by De Preter et al. (2007) [30] was a parallel control trial and combined a pre- and probiotic that had both shown significant benefits on uremic toxin reduction in isolation [30]. This synbiotic intervention found a significant reduction in urinary PC with a tendency for an additive effect beyond that seen in the individual benefit of pre- and probiotics. De Preter et al. (2006) administered a prebiotic at a dose known to reduce urinary PC levels, 20 g lactulose, together with a probiotic that had previously shown no effect, *Saccharomyces boulardii*. Interestingly this intervention resulted in a less pronounced effect compared to the prebiotic in isolation [28]. The authors hypothesised that the cause of this less pronounced effect may have been due to the yeast probiotic being capable of using the prebiotic as an energy source leaving less available for the colonic microbiota.

The potential risk of publication bias was indicated in the funnel plot, where only two negative interventions were found in the literature search [28, 31]. Swanson's et al. study found no effect in pre-, pro-, or synbiotic groups at the end of the four-week supplement period [31]. This negative study found a surprising result where the concentrations of PC, IS, and nearly all fecal parameters increased from week two to week four, for all intervention groups and placebo. This study included some dietary monitoring and reported that there was no significant difference in protein intake that could have explained this trend.

Production of PCS and IS is reliant on the amount of protein that enters the large intestine. Exogenous (dietary) protein is the primary source of metabolised protein. There are a number of factors which alter the amount of protein that reaches the large intestine, escaping digestion in the small intestine. This includes protein form, that is, cooked or uncooked, and protein source, that is, animal or plant, as well as several gastrointestinal abnormalities that have been reported in uremic patients including gastrointestinal mobility disorders, small-bowel bacterial overgrowth, gastric hypochlorhydria, and pancreatic abnormalities [37, 38].

It is well established that restriction of dietary protein decreases the generation of both PCS and IS [3]. Several human studies have shown that increases in dietary protein result in increased serum, urine, and/or fecal concentration of PCS and/or IS [39–41]. This highlights that diet is a major confounder to assessing the real benefit of pre- and probiotics on PCS and IS production. Thereby controlling for dietary intakes is of importance in elucidating the effect of these interventions; however, this was rarely undertaken by studies featured in this paper.

It has been recognised that the preintervention values of both the uremic toxins and microflora are correlated with the effect size of pre- and probiotic interventions [18, 19]. This was highlighted in the study by De Preter et al. [19] which found a significant correlation between the baseline levels of urinary PC and effect size of the prebiotic intervention.

The majority of the studies looking at the effect of pre- and probiotics have been conducted in the healthy population where levels of these toxins are low and the microflora is “normalised.” The purpose of reviewing these studies was to confirm the mechanism of PCS and IS reduction through pre- and probiotics. Given that the CKD population have both lower *Bifidobacterium* and higher uremic toxin levels, the effect size in this group is expected to be significantly higher. It is important to note, however, that the concentration of the uremic toxins differs not only between the different populations, that is, HD and healthy, but also within the HD populations studied. This is attributed to a number of factors including cultural food intake, presence of diabetes, and residual renal function [39, 42]. The common use of antibiotics in the HD population is another factor known to influence the gut microflora and could be attributed to variations in the production of these toxins [43]. It is for this reason that most of the studies reported exclusion criteria around antibiotic use within at least two weeks of study commencement.

For both pre- and probiotics, there appears to be a threshold dose required to see a benefit and beyond this dose there is no additive benefit. Comparing across studies the three interventions which used at least 20 g of lactulose [19, 28, 29] found a decrease in PCS whereas the study which used only 3g per day did not [22]. Studies showed no difference in effect size with daily doses between 20 and 30 g of lactulose, [28] 7.5 and 15 g of trans-GOS [24], and 3 × 10^9^ and 12 × 10^9^ of *Bifidobacterium* longum [34]. 

The duration of supplementation further complicates the concept of a threshold dose. A *Lactobacillus* probiotic administered at the highest dose in this paper, 1 × 10^11^, for one week found no significant change in IS, whereas another *Lactobacillus* probiotic administered at a 2-fold lower dose over one month resulted in a significant change. 

Given the diversity in the survival rates of different probiotic strains, different characteristics within prebiotic varieties, namely, their different bifidogenic capacities [19], and the minimum duration of supplementation, it is not possible to set a universal threshold dose for either pre- or probiotics. 

There was no prebiotic effect seen on the oral-caecal transit time despite this being a recognised benefit of several prebiotics and a potential mechanism for lowering the amount of amino acid fermentation in the large intestine [43, 44]. However, this was tested in only four of the healthy population studies, none of which had any GI issues, compared to the HD population where constipation is common [45]. There was also no change in fecal biomass measured as fecal weight. This is in line with the recent literature which supports that prebiotics alter the type of bacteria but not the total number [23]. Prebiotic-induced flatulence was reported, though there was insufficient data to determine a dose-dependent effect [18, 24, 32]. All efforts were made to ensure this symptom was controlled including multiple smaller doses over the day. The literature also suggests that the increase in flatulence is transient and may resolve over time [46, 47].

There were a number of findings from the microbiota analysis which further support the mechanistic role of pre- and probiotics in reducing PCS and IS. Most of the microbiota analysis was undertaken in the prebiotic interventions with a focus on changes in bifidobacteria, a common property of prebiotics. Two of these studies saw a reduction in fecal PC and indole, and along with increases in *Bifidobacterium* and *Lactobacillus* there was a decrease in Bacteroidaceae [20, 22]. The Bacteroidaceae family includes a main producer of PCS [25]. Therefore the combination of the decrease in PCS producing bacteria and the increase in PCS and IS repressing bacteria (*Bifidobacteria* and *Lactobacillus*) resulted in significant improvements [25, 34].

Only one probiotic study provided a detailed investigation of the effect on the microbiota [35]. This study illustrated that the fecal flora in the HD population before intervention contained a significantly greater proportion of aerobic bacteria (specifically *Escherichia coli*), 100 times higher than that in healthy matched controls, and significantly lower *Bifidobacteria*. Following probiotic administration in the HD population, this study observed a significant decrease in the enterobacteria along with a decrease in serum IS levels. As *Escherichia coli* has one of the highest observed tryptophanase activities (the enzyme that produced IS), this is a clear example of one of the mechanisms of probiotics.

These findings not only support the potential role that pre- and probiotics may play but also emphasise the need to selectively choose probiotic strains and prebiotic varieties that inhibit the production of bacteria which aid mechanistic association with IS and PCS production.

A number of the studies evaluated in this paper were considered to have no effect, or did not reach statistical significance following pre-, pro-, or synbiotic intervention. Within these studies, several common limitations were identified, including short study duration [27], lower baseline levels of the toxin [32], small prebiotic doses of pre- and/or probiotics [21, 22, 35], small sample size [23], or did not find a decrease in bifidobacterium levels [24].

There are two other therapeutic possibilities for reducing IS and PCS: a low protein diet and oral charcoal adsorbent AST-120. Low protein diets may be contraindicated in the CKD population, especially in the dialysis population with high protein requirements, along with the increased risk of malnutrition, influence on quality of life, and adherence concerns [48–50]. Oral charcoal adsorbent AST-120 (Kremezin, Kureha Chemical Industry, Tokyo, Japan) has been more recently investigated as an effective agent for preventing intestinal absorption of both indole and PC. This compound completed Phase III investigations in the USA in November 2011 [51], and previously demonstrated in earlier studies in Asian countries a delay in the progression of CKD [6]. The side effects of AST-120 are not insignificant, as it may absorb other beneficial nutrients along with uremic toxins, result in constipation and GI upset, and require large fluid intake, all of which are potentially contraindicated in this population group [35].

Nevertheless, AST-120 studies have demonstrated significant decreases in IS. This has been subsequently associated with improvements in CV markers, such as carotid intermedial thickness, arterial stiffness (pulse wave velocity) [52] and flow-mediated vasodilatation [53], and postponement of the start of dialysis [54–56]. Given that pre- and probiotic treatment in CKD is in its infancy, there is a lack of studies measuring its effect on clinical outcomes. Fortunately this benefit can be extrapolated from the AST-120 studies which also measure serum IS concentrations with similar baseline concentrations. The effect size in IS reduction, resulting from AST-120 administration, of 2.8 mg/L resulted in an increase in flow-mediated dilatation in the brachial artery (endothelial function) [53] and a 5.5 mg/L reduction in IS delayed CKD progression [57]. This effect size in IS reduction is in line with the reduction achieved with pre- and probiotics in the HD population illustrated in the meta-analysis (6.4 mg/L).

## 5. Conclusion

Altering the microbiota via pre- and/or probiotics is a potential treatment for reducing bacterial protein fermentation and therefore the generation of PCS and IS, two nephro- and cardiovascular toxins. This investigation demonstrates that pre-, pro-, and synbiotics hold great potential in lowering PCS and IS production in the CKD population, which may potentially be translated into benefits to clinical outcome, such as reduction in CVD markers and CKD progression. This paper illustrates from the 19 eligible studies looking at this intervention on PCS and/or IS reduction that there is a positive trend for both pre- and probiotics. Unfortunately, there are a number of confounders that hinder the evaluation of this treatment. Strict control of dietary intake as well as appropriate selection of probiotic strains and prebiotic varieties is of importance. The increasing prevalence of CKD coupled with high mortality and morbidity rates and treatment costs presents a compelling and urgent need for further investigation into a cost-effective treatment such as pre- and probiotics. Future well-designed studies are needed so that the full potential of this treatment can be uncovered supporting its application in the clinical setting.

## Figures and Tables

**Table 1:** Published studies on the effectiveness of prebiotics on reducing indoxyl sulphate and p-cresyl sulphate.

Author (year)	Study design	Patients	Supplement total dose/day (CFU or g) number of doses/day	Duration	Analysis method	Main results (preintervention and postintervention (mean difference (SD)))	Comments	Grade benefit
Cloetens et al., (2010) [18]	Randomised-placebo-controlled crossover trial	*n* = 20 healthy (6 male)	Prebiotic: AXOS degree of polymerization = 6 10 g administered in orange juice Placebo: maltodextrin 10 g 2 dose/day	3 wks in each arm	Heat and acid deproteinisation, GC, MS	Δ *Urinary PC (mg/24 h) * ^*∧*^ (within group, 0–2 wks) Tx grp: 32.1–14.9 (−17.2 ) (0.011) Placebo: 32.9–27.3 (−5.6 ) (ns) (within group, 0–3 wks) Tx grp: 32.1–27.2 (−4.9) (ns) Placebo: 28.2–25.2 (−3.0) (ns)	(i) 4-week washout period between each arm (ii) Analysis 0–2 weeks *n* = 10 only in both groups (iii) Analysis at week 3 was taken the day after prebiotic was ceased (iv) Increased bifidobacterium levels at 2 weeks, *P* = 0.025; compared to placebo, this was lost at week 3 (v) Correlation pre- and postintervention bifidobacterium levels *r* = −0.51 *P* = 0.022 (vi) Usual diet, asked to have a regular eating pattern (3 meals/day), and foods containing prebiotics were limited to 1/week	Moderate PC + (0–2 wks)

De Preter et al., (2008)[19]	Case series (2 independent studies—S1 and S2)	*n* = 48 healthy S1, *n* = 29 (14 male) S2,*n* = 19 (9 male)	S1: lactulose 20 g S2: OF-IN (Orafti Synergy1) (i) Oligofructose degree of polymerization = 4, 10 g (ii) Raftiline HP degree of polymerization = 25, *β*(2,1) linkage, 10 g 2 dose/day	0–4 wks	Heat and acid deproteinisation, GCMS	Δ *Urinary PC (mg/24 h)*(wk 0–4) S1: 20.7–12.7 (−8) (0.001) S2: 27.7–17.8 (−9.9) (0.005)	(i) Both studies increased in bifidobacterium levels S1: *P* = 0.017, S2: *P* < 0.001, and no difference between studies (ii) Correlation between baseline PC levels and effect of prebiotic intervention (*r* = −0.64) (S1), *r* = −0.74 (S2) *P* < 0.001) (iii) No runout period (iv) Usual diet, advised to keep stable. Avoid fermented milk products and foods containing fermentable carbohydrates	Low PC +

Ito et al., (1993a) [20]	Case series	*n* = 12 healthy (all male)	TD (including GOS) 15 g administered in iced tea 1 dose/day	0–6 days	Heat and acid deproteinisation GC, FID	Δ *Fecal (ug/g wet wt)*(day 0–6) PC 9.8–3.3 (−6.5) (<0.05) Indole 10.5*–*7.4 (−3) (<0.01)	(i) Post was measured on the 6th day of ingestion (ii) Increased bifidobacterium levels and lactobacillus *P* < 0.01 and *P* < 0.05, respectively. Decrease in bacteroidaceae *P* < 0.05 (iii) 6 day runout levels PC, indole returned towards baseline (iv) Usual diet with no lactose containing foods or fermentation products	Low PC+ IS+

Ito et al., (1993b) [21]	Case series	*n* = 12 healthy (all male)	GOS (Oligomate 50) 2.5 g administrated in apple juice which included 1 dose/day	0–3 wks	Heat and acid deproteinisation GC, FID	Δ *Fecal (mg/L)* (wk 0–3) Indole 50.4*–*31.6 (−18.8) (<0.05) PC 48.7–41.1 (−7.6) (ns)	(i) Post was measured on 6th day of week 3 (ii) Participants were selected out of a group of 28 on the basis of the lowest bifidobacterium levels (iii) Increased bifidobacterium levels *P* < 0.05 (iv) 6 day runout levels indole decreased further 23.4 mg/L (v) Usual diet with no lactose containing foods or fermentation products	Low IS+ PC−

Terada et al., (1992) [22]	Case series	*n* = 8 healthy (*n* = 5 male)	Lactulose 3 g administered in a drink 1 dose/day	0–2 wks	Heat and acid deproteinisation GC, FID	Δ *Fecal (mg/d)* (wk 0-1) Indole 6.3–2.4 (−3.9) (<0.05) PC (not reported) (wk 0–2) Indole 6.3–0.9 (−5.4) (<0.05) PC (not reported) (ns)	(i) Increased bifidobacterium levels and lactobacillus *P* < 0.001 and *P* < 0.05, respectively. Decrease in bacteroidaceae *P* < 0.05 (ii) Usual diet. Avoided food with abundant viable cultures (iii) 1 week runout period levels returned to preintervention levels*	Low IS+ PC−

Geboes et al., (2006) [23]	Case series	*n* = 7 healthy (*n* = 2 male)	Inulin degree of polymerization = 12, *β*(2,1) linkage 15 g 3 dose/day	0–4 wks	Heat and acid deproteinisation, GCMS	Δ *Urinary PC (mg/24 hr) * ^*∧*^ (wk 0–4) Total: 32.3–22.3 (−10) (ns) Percentage of isotope ([2H4]p-cresol): 0.81–0.51 (−0.3 ) (ns)	(i) Low overall recovery of the label (ii) 1 week runout urinary PC and urinary percentage of isotope increased towards baseline. Fecal PC exceeded baseline, 13.33 mg/72 hrs, and fecal percentage of isotope decreased further to 0.44%	Very low PC−
					Outcome markers-total PC and stable isotopes [2]p-cresol given as [2]tyrosine in test meal	Δ *Fecal PC (mg/72 hr) * ^*∧*^ (wk 0–4) Total: 9.7–9.3 (−0.4) (ns) Percentage of isotope ([2H4]p-cresol): 0.78–0.71 (−0.07) (ns)	(iii) Usual diet, advised to keep constant macronutrient composition. Diet records kept at intervals throughout the study to allow for qualitative comparison—results not reported	

Alles et al., (1999) [24]	Placebo controlled nonrandomised experimental trial	*n* = 39 healthy High dose = 14 (8 male) Low dose = 13 (7 male)	TOS degree of polymerization = 2, Elix' or high dose group = 15 g	0–3 wk standardise run in diet	Freeze-thaw deproteinisation, HPLC, UV	Δ *Fecal indole (ug/g wet wt) *(mean difference (95% CI)) (between placebo and intervention wk 3–7)	(i) Intervention and placebo increased in bifidobacterium levels.	Low
		Placebo = 12 (7 male)	Low dose group = 7.5 g Administered in juice	3–7 wk intervention		High dose group: −3.0 (−11,6) (ns)	(ii) No run-out period	
			3 dose/day			Low dose group: −5.0 (−11,6) (ns)	(iii) 90% of food was provided according to a dietitian prescribed standardized low-fibre high-protein diet	IS−

Meijers et al., (2009) [32]	Case series	*n* = 22 hemodialysis (15 male)	OF-IN (Orafti Synergy1) total 20 g	0–4 wks	Sodium octanoate, HPLC, FS	Δ *Serum (mg/L) * ^*∧*^	(i) Commenced 1/2 dose: 1/day first wk, increase to full by week 2	Very Low
			(i) Oligofructose degree of polymerization = 4, 10 g			wk 0–4		
			(ii) Raftiline HP degree of polymerization = 12, *β* (2,1) linkage, 10 g			PCS 38.5–32.0 (−6.5) (0.01)	(ii) PCS levels remained significantly different 4 weeks post	PC+
			2 dose/day			IS 23.7–22.4 (−1.3) (ns)	(iii) No monitoring of diet	IS–

**Table 2:** Published studies on the effectiveness of probiotics on reducing indoxyl sulphate and p-cresyl sulphate.

Author (year)	Study design	Patients	Supplement total dose/day (CFU or g) number of doses/day	Duration	Analysis method	Main results (preintervention and postintervention (mean difference))	Comments	Grade benefit
Ling et al., (1994) [25]		*n* = 33 healthy female	*Lactobacillus rhamnosus* GG 1 × 10^11^ administered in yogurt	0–4 wks	Heat and acid deproteinisation, HPLC, FS	Δ*Urinary PC mg/24 hr *	(i) Subgroup from a larger study	Moderate
	Placebo-controlled non-randomised experimental trial	*n* = 22 treatment				(within group, wk 0–4)	(ii) 2 week runout period levels increased towards baseline levels 41.5 mg/24 hr	
		*n* = 11 placebo	1 dose/day			Tx grp: 45.0–35.5 (−9.5) (<0.05)	(iii) Both groups were also given 9 g fibre from aleuronic layer of whole-grain rye daily	PC+
						Placebo: 46.5–43.1 (−3.4) (ns)	(iv) 3 day food records were analysed for macro nutrients and fibre questionnaire for total fibre intake. No significant difference between groups was reported	

Tohyama et al., (1981) [26]		*n* = 7 healthy (all male)	*Lactobacillus casei* 90241 1 × 10^10^ administered in milk	0–3 wk control 4–9 wk intervention	Heat and acid deproteinisation, GC, FID	Δ*Urinary (percentage of reduction (±SD)) *	(i) Urine was analysed at different times of the day between baseline and post	Very Low
	Interrupted time series without a parallel control group					(within group, wk 4–9)	(ii) Included rat study which showed significant reductions also	
						PC − 42.6 (±33.7) (<0.05)	(iii) Strong correlation between fecal tryptophanase activity and urinary IS (*r* = 0.93)	
			1 dose/day			IS − 29.3 (±15.9) (<0.05)	(iv) 2 week run-out period concentrations returned to initial levels post feeding—data not shown	PC+
							(v) No dietary restrictions	IS+

Fujiwara et al., (2001) [27]	Case series	*n* = 8 healthy	*Lactobacillus gasseri* SBT2055SR-lyophilized 1 × 10^11^ administrated in milk 1 dose/day	0-1 wk	Deprotinisation method was not disclosed, HPLC, UV-VIS	Δ*Fecal mg/g* (wk 0-1) PC 0.064–0.022* (− 0.045) (0.01) Indole (not reported) (ns)	(i) Subgroup of another study (ii) Decreased staphylococcus *P* < 0.05 (iii) By 5 day run-out PC levels returned to baseline* (iv) Usual diet with restriction on fermented milk, pickles, and natto	Low PC+ IS−

Takayama et al., (2003) [33]	Nonrandomised-placebo controlled experimental trial	*n* = 22 hemodialysis (*n* = 14 male)	*Probiotic*: *Bifidobacterium longum* strain JCM008 administered in gastroresistant capsules 3 × 10^9^	0–5 wks	Deproteinization (not disclosed) Reverse-phase HPLC, FS	Δ*Serum IS (mg/L)* (within group, wk 0−5)	(i) Placebo has different dose and strain	Low
		*n* = 11 treatment	*Placebo*: Bifidobacterium (powder form) 2 × 10^7^			Tx group: 49.0–35.0 (−14.0) (<0.005)	(ii) 2 week runout period levels increased towards baseline levels 44 mg/L	IS+
		*n* = 11 placebo	Dose/day not disclosed			Placebo: 48.0–52.0 (+4.0) (ns)	(iii) No monitoring of diet	

Taki et al., (2005) [34]		*n* = 27 hemodialysis (*n* = 14 male)	*Bifidobacterium longum *	0–12 wks	Not disclosed	Δ*Serum IS (mg/L) *	(i) Strain not disclosed	Very low
			0–4 wks: 3 × 10^9^			(wk 0–4) 35.1–31.0 (−4.1) (<0.01)	(ii) Analysis methods not described	
	Case series		4–8 wks: 6 × 10^9^ 8–12 wk: 12 × 10^9^			(wk 0–8) 35.1–31.7 (−3.4) (<0.05)	(iii) No dose response effect	
			Administered in gastroresistant capsule			(wk 0–12) 35.1–31.9 (−3.2) (<0.05)	(iv) No runout period	
			Dose/day not disclosed				(v) Monitoring of diet (not disclosed)	IS+

Hida et al., (1996) [35]		*n* = 20 hemodialysis (*n* = 8 male)	*Bifidobacterium infantis*, *Lactobacillus acidophilus*, *Enterococcus faecalis* 2 × 10^8 ^of each strain administered in a capsule	0–4 wks	Plasma: reverse-Phase HPLC, UV detection	Δ*Plasma (mg/L)* (wk 0–4)	(i) Strain not disclosed	Very low
	Case series				Fecal: steam distilled, GC, FID	IS 45.2–31.1 (−14.1) (<0.01)	(ii) Fecal analysis included 10 patients only from 0–2 week	IS+
						PC 17.8–18.3*(+0.5) (ns)	(iii) Decreased enterobacteria *P* < 0.05	PC+ (fecal)
			2 dose/day			Δ*Fecal (mg/g)* (wk 0–4) PC 102.0–70.0* (−32.0 ) (<0.01)	(iv) No runout period	PC− (serum)
						Indole 45.0–32.0* (−13.0) (<0.05)	(v) No monitoring of diet	

**Table 3:** Published studies with more than one intervention (Pre-, Pro- and/or Synbiotics) on reducing indoxyl sulphate and p-cresyl sulphate.

Author (year)	Intervention	Study design	Patients	Supplement total dose/day (CFU or g) number of doses/day	Duration	Analysis method	Main results (preintervention and post intervention (mean difference))	Comments	Grade benefit
De Preter et al., (2006) [28]	Prebiotic Probiotic Synbiotic (Group3)	Randomised placebo controlled cross over trial	*n* = 43 healthy (*n* = 21 male) Group 1 : 14 Group 2 : 14 Group 3 : 15	Probiotic: lyophilized *Saccharomyces boulardii* A07FA02 (yeast) administered via capsules Group1 = 2–5 × 10^9^ Group2 = 4–10 × 10^9^ Group3 = 2–5 × 10^9^ Prebiotic: lactulose Group1 = 20 g Group2 = 30 g Group3 = 20 g Placebo: maltodextrin 2 dose/day	4 wk in each arm Probiotic Prebiotic Placebo (Group 1 and 2) Synbiotic (Group3)	Heat and acid deproteinisation, GC, MS	*Urinary PC (mg/24 hr) *(between placebo and intervention at 4 wk) Prebiotic arm Group1: 17.9, 9.5 (−8.4) (0.022) Group2: 21.9, 14.7 (−7.2) (0.022) Probiotic arm Group1: 17.9, 21.0 (+3.1) (ns) Group2: 21.9, 18.4 (−3.5) (ns) Δ*Urinary PC (mg/24 hr)*(within group, 0–4 wks) Prebiotic arm Group1: 17.7–9.5 (−8.2) (0.019) Group2: 20.1–14.7 (−5.4) (0.002) Group3: 20.2–16.2 (−4.0) (0.002) Probiotic arm Group1: 17.7–21.0 (+3.3) (ns) Group2: 20.1–18.4 (−1.7) (ns) Group3: 20.2-20.2 (0.0) (ns) Synbiotic arm Group3: 20.2–18.9 (−1.3) (ns)	(i) Variability of probiotic dose per capsule (1–2.5 × 10^9^) (ii) Δ Fecal concentration was also assess; however, no trend was evident among the three groups (iii) 4 week run-out period levels returned towards baseline becoming statistically significant compared to week 4 (iv) Usual diet, advised to keep stable. Avoid intake of fermented milk products and food components containing high quantities of fermentable carbohydrates	Moderate PC+ (prebiotic) PC– (probiotic)

De Preter et al., (2004) [29]	Probiotic Prebiotic	Randomised placebo controlled cross over trial (2 independent studies: probiotic and prebiotic)	*n* = 19 healthy Probiotic, *n* = 10 Prebiotic, *n* = 9	Probiotic: *Lactobacillus casei Shirota* 13 × 10^9 ^ administered in milk product Placebo: milk product without strain Prebiotic: lactulose 20 g Placebo: lactose	2 wks in each arm Probiotic Prebiotic Placebo	Heat and acid deproteinisation, GC, MS Outcome markers-total PC and stable isotopes [^2^H_4_]p-cresol given as [^2^H_4_]tyrosine in test meal	Δ*Urinary PC (mg 0–24 hr) *(difference in intervention Group, difference in placebo (mean difference) 0–2 wk) Probiotic study Total: 6.6, 8.4 (−1.8) (ns) Percentage of isotope: 0.10, 0.25 (−0.15) (ns) Prebiotic study Total: −11.2, 8.3 (−19.5) (0.018)	(i) Theory-based explanation of different phases of total urinary PC, that is, 0–24 hr and 24–48 hr; no other paper measures PC in this way (ii) Low overall recovery of the label (iii) 2-week washout period in between each arm (iv) Usual diet, advised to keep stable. Avoid intake of fermented milk products and food	Moderate PC+
							Percentage of isotope: −0.87, 0.05 (−0.92) (0.005)	components containing high quantities of fermentable carbohydrates	
				2 dose/day			Δ*Urinary PC (mg 24–48 hr)* (difference in intervention group, difference in placebo (mean difference) 0–2 wk) Probiotic study Total: −16.2, 8.3 (−24.5) (0.009) Percentage of isotope: −0.70, 0.46 (−1.16) (0.042) Prebiotic study Total: 8.9, 6.6 (+2.3) (ns) Percentage of isotope: −0.72, −0.10 (+0.28) (ns)		

De Preter et al., (2007) [30]	Prebiotic Probiotic Synbiotic	Randomized placebo-controlled crossover trial	*n* = 19 healthy (*n* = 10 male) Group 1 : 10 Group 2 : 9	Probiotic: Group 1—lyophilized *Bifidobacterium breve* Yakult 2 × 10^9^ Group 2—*Lactobacillus casei Shirota* 13 × 10^9^ administrated in milk product Prebiotic: Group 1 and 2—OF-IN (Orafti Synergy1) total 20 g (i) Oligofructose degree of polymerization = 4, 10 g (ii) Raftiline HP degree of polymerization = 12, β (2, 1) linkage, 10 g Placebo: strain free milk product (probiotic)/ Maltodextrine (prebiotic)	Each Group 4 weeks in each arm Probiotic Prebiotic Placebo Synbiotic (Group 2 only) 0–4 wks	Heat and acid deproteinisation, GC, MS	Δ*Urinary PC (mg/24 h) * ^*∧*^ (within group, 0–4 wks) Probiotic: Group 1: 21.2−16.7 (−4.5) (0.005) Group 2: 24.4–20.5 (−3.9) (0.038) Prebiotic Short-term effect (start of study) Group 1: 21.2–15.7 (−5.5) (0.013) Group 2: 24.4–14.7 (−9.7) (0.025) Long-term effect (end of study, wk4) Group 1: 21.2−21.3 (+0.2) (ns) Group 2: 24.4–13.4 (−11.0) (0.025) Group 1 + 2: (0.005) Synbiotic: Group 2: 24.4–9.8 (−14.6) (0.021)	(i) Analysis at week 4 was taken the day after prebiotic was ceased (ii) Increased bifidobacterium levels after prebiotic intervention *P* = 0.006 (iii) Analysis based on baseline result at week 0 and not baseline of each period following washout (iv) 2-week washout period in between each arm; values increased during this time (except placebo)	Low PC+
							Placebo Group 1: 21.2–22.0 (+0.8) (ns) Group2: 24.2–25.8 (+1.6) (ns)	(v) Usual diet, advised to keep stable. Avoid intake of fermented milk products and food components containing high quantities of fermentable carbohydrates	

Swanson et al., (2002) [31]		Randomised placebo controlled trial	*n* = 62 healthy (*n* = 25 male)	Probiotic: free-dried *Lactobacillus acidophilus* NCFM powder ≥ 2 × 10^9 ^administered in hard gelatin capsules coated with acid resistant chemical Placebo: cornstarch	0–4 wks	Freeze thaw deproteinisation, GC, FID	Δ*Fecal (mg/g dry matter) *(within group, wk 0–4)	(i) Concentration of all fecal parameters increased from week 2–4 even in the control group	Very low
	Probiotic Prebiotic Synbiotic		Placebo, *n* = 15 Prebiotic, *n* = 15 Probiotic, *n* = 15	Prebiotic: fructose oligosaccharide, (Nutraflora) 6 g administered in non carbonated beverage Placebo: sucrose			Probiotic Indole 0.13–0.18 (+0.06) (ns)	(ii) Conclusions were made base on nonstatistically significant data	
			Synbiotic, *n* = 17	2 dose/day			PC 0.26–0.30 (+0.04) (ns) Prebiotic Indole 0.10–0.11 (+0.01) (ns) PC 0.23–0.23 (0) (ns) Synbiotic Indole 0.09–0.08 (−0.01) (ns)	(iii) No runout period (iv) 3 day food records were analysed for macro nutrients and fibre pre-, during, and postintervention.	PC– IS–
							PC 0.21–0.26 (+0.05) (ns) Placebo Indole 0.12–0.13 (+0.01) (ns) PC 0.22–0.19 (−0.03) (ns)	(v) Substantial difference in total fibre intakes, that is, probiotic group had 10 g more than prebiotic group at week 6	

Nakabayashi et al., [36] (2011)	Synbiotic	Interrupted time series without a parallel control group	*n* = 7 hemodialysis	Probiotic (i) *Lactobacillus casei* strain Shirota,	0–2 wk runin	Heat acid deproteinization, HPLC, FS	Δ*Serum (mg/L) * ^*∧*^ (within group, wk 2−4)	(i) 1 participant used medications that contained live lactic acid bacteria	Very Low
				(ii) *Bifidobacterium breve* strain Yakult administered in powder form 3 × 10^8^ each strain	3–5 wk intervention		PC 17.1–14.2 (−2.9) (0.031)	(ii) No runout period	PC+
				Prebiotic: GOS (oligomate 55 N) ≥5 g 3 dose/day			IS 32.2–30.1 (−1.8) (ns)	(iii) No monitoring of diet	IS–

Key:

^*∧*^median difference.

*estimated value from graph, exact value not reported.

^#^paper stated no change, exact figures not reported.

^@^no trend was evident among three groups.

AXOS: arabinoxylan-oligosaccharide; FID: flame ionisation detection; FS: fluorescence spectroscopy; GC: gas chromatography; GOS: galactooligosaccharide; HPLC: high performance liquid chromatography; IS: indoxyl sulphate; MS, mass spectrometry; OF-IN, oligofructose-enriched inulin; PC/S: p-cresyl/sulphate; SD: standard deviation; TD: transgalactosylated disaccharide; TOS: trans-galaco oligosaccharide; Tx Grp, treatment group; UV: ultra violet; VIS: visible; wk: week.
